# Resilience Phase Transitions in Networks with Heterogeneous Node Growth Rules

**DOI:** 10.3390/e28101135

**Published:** 2026-10-15

**Authors:** Wanjing He, Yufeng Zhuang, Yanzhu Hu

**Affiliations:** School of Intelligent Engineering and Automation, Beijing University of Posts and Telecommunications, Beijing 100876, China; hewanjing.220126@bupt.edu.cn (W.H.); bupt_automation_safety_yzhu@bupt.edu.cn (Y.H.)

**Keywords:** complex networks, structural robustness, heterogeneous attachment, network fragmentation, degree entropy

## Abstract

Network growth and node-removal robustness are often compared while node arrivals and link budgets also vary. We hold these two quantities fixed using the email-Eu-core and CollegeMsg temporal communication networks and vary only the selection of older attachment targets. Uniform, degree-only, activity-only, combined degree-activity, and hybrid kernels are evaluated with 30 independent graph realizations per setting. Random failures use 50 deletion orders per graph; intentional attacks use static degree, exact static betweenness, and degree recalculated after every deletion. Uncertainty is summarized by realization-level 95% confidence intervals. Under static degree attack, the mean integrated robustness falls from 0.456 to 0.313 for email-Eu-core and from 0.388 to 0.176 for CollegeMsg when uniform attachment is replaced by the combined kernel. Both single-factor kernels are less damaging than their combination, and the ranking of the five counterfactual rules is unchanged under adaptive degree removal. The combined kernel has higher intact-network global efficiency but lower random-failure robustness than uniform attachment in both data sets; no universal reversal between random and intentional attacks is observed. Susceptibility peaks are finite-network, grid-dependent fragmentation indicators, not estimates of thermodynamic critical points. The results identify how retrospective target-selection counterfactuals redistribute structural vulnerability and separate this effect from dynamical recovery.

## 1. Introduction

The topology of a network constrains the damage caused by component loss. Early analyses of complex networks established that heterogeneous degree distributions can coexist with tolerance to random failures and vulnerability to hub removal [1,2,3]. That contrast is useful, but it is not a universal ranking theorem: attack protocol, finite size, degree correlations, and the normalization of the response all matter. Recent reviews distinguish structural robustness from dynamical resilience and recovery [4,5,6,7,8]. We use structural robustness to mean retained undirected connectivity during node deletion; we do not model recovery or adaptation of node states.

In the title, resilience refers specifically to structural robustness, and phase transitions refers to percolation-like fragmentation in finite networks. The susceptibility peaks examined here are operational indicators on a discrete removal grid, not evidence of a thermodynamic singularity. No finite-size scaling or dynamical recovery analysis is performed.

Growth determines which nodes acquire the links that later sustain connectivity. Preferential attachment emphasizes cumulative advantage [9], whereas fitness-based growth allows intrinsic attributes to influence target selection [10]. In a communication network, observed activity is another plausible correlate of link opportunity. A kernel that multiplies degree and activity therefore combines two mechanisms. Comparing that kernel only with uniform attachment cannot identify the separate effects of its factors.

A further obstacle is experimental comparability. Changing the growth process often changes the number of nodes, the number of edges, or the sequence of opportunities to acquire links. We instead retain the empirical ordering of first-observed nodes and the number of distinct older neighbors associated with each node. Target identities are then reassigned under alternative kernels. This is a retrospective structural comparison conditional on an observed history, not a causal reconstruction of individual communication decisions.

Degree-distribution entropy provides a complementary description of the resulting topology, but its interpretation requires care. Entropy over degree classes measures diversity among occupied classes, not the equality of individual node degrees. A regular graph can have zero degree-class entropy despite perfectly equal degrees. We therefore interpret entropy jointly with the degree Gini coefficient, maximum degree, and intact-network efficiency rather than treating entropy as a stand-alone robustness score.

This study asks three questions. First, how do degree and activity preferences separately and jointly change structural robustness under a fixed empirical link budget? Second, do the comparisons persist when an attacker updates degree rankings after every deletion? Third, how sensitive are the conclusions to mixing strength, activity sensitivity, numerical grid, and centrality approximation? The analysis uses two temporal data sets, five principal kernels, a continuous-parameter sweep sampled on a grid, and explicit Monte Carlo uncertainty.

## 2. Materials and Methods

### 2.1. Data and Retrospective Arrival Constraints

**Figure 1:**
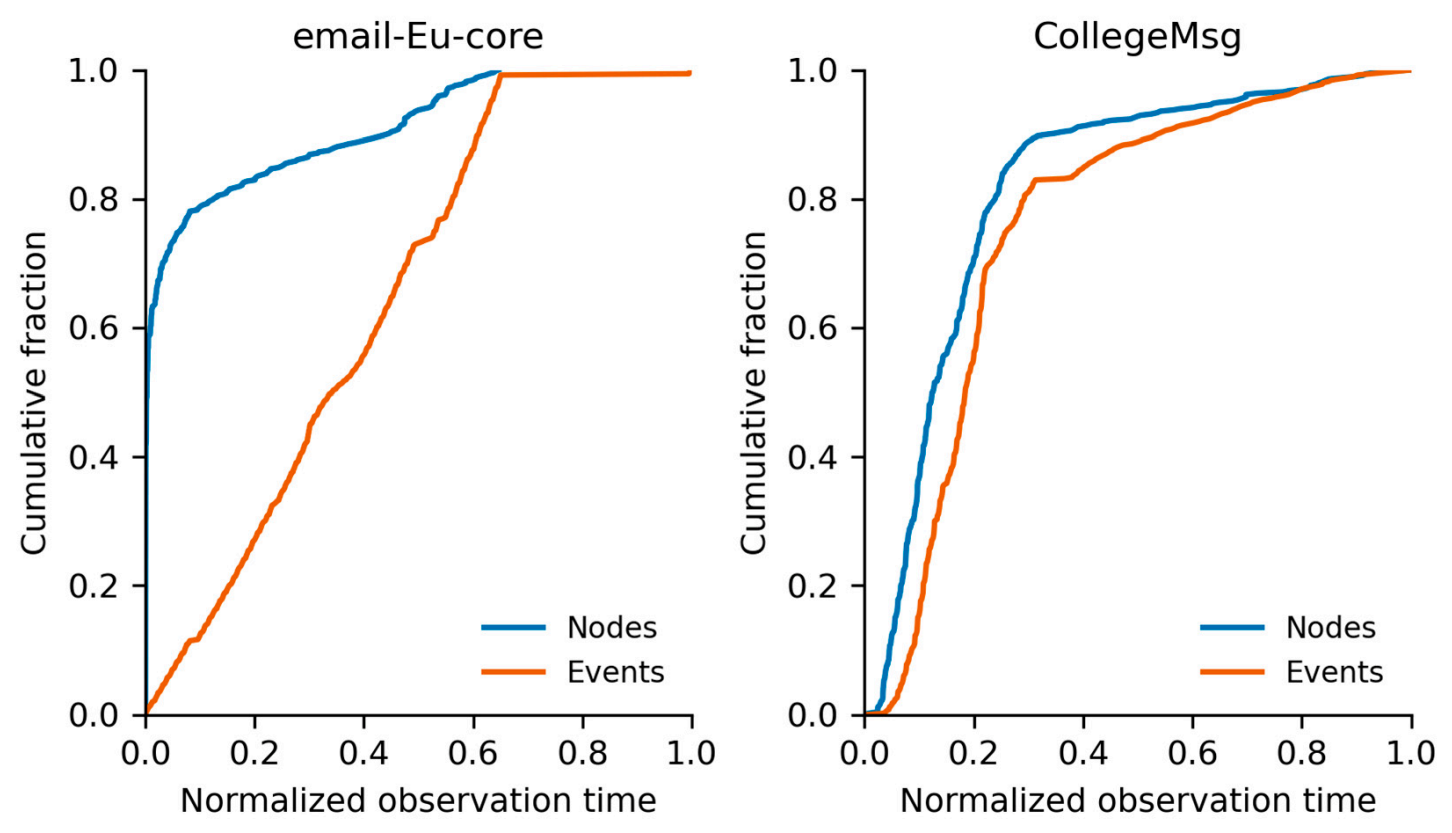
Cumulative node arrivals and interaction events, normalized by their final counts and the observation duration. These curves describe the input data, not counterfactual simulation outcomes.

We analyze the email-Eu-core temporal network and the CollegeMsg network from the Stanford Large Network Dataset Collection (SNAP) [11,12]. Each record gives a sender, a recipient, and a timestamp. We remove self-loops, retain repeated interactions when measuring activity, and collapse distinct dyads to undirected edges for connectivity analysis. Nodes are ordered by their first incident event; ties are resolved by ascending original integer node identifier. Table 1 reports the retained data, and Figure 1 shows cumulative arrivals and interactions.

Let N be the number of retained nodes and M the number of unique undirected edges. Nodes are indexed from 0 to N−1. For arriving node i, the budget $m_{i}$ is the number of its empirical neighbors with smaller indices. Every empirical edge is counted once at its later endpoint, so the sum of these budgets is M. Some budgets may be zero. Reassigning targets preserves each budget exactly, not merely the average degree.

These constraints use the complete observation window. An edge assigned to a node at its conceptual arrival may have been observed much later, and empirical activity also uses the full record. Consequently, the counterfactual does not preserve individual edge timestamps, directed paths, or time-respecting communication routes. Time-varying network and dynamical resilience studies address related but different questions [13,14]. The present comparison concerns final structural consequences under a retrospective arrival budget.

### 2.2. Attachment Kernel and Multi-Link Sampling

For each arriving index i=1,...,N−1, there are exactly i older candidates, indexed j=0,…,i−1. Their degrees $k_{j}$ are measured in the partially constructed undirected graph. Let $a_{j}$ be the number of retained events incident on node j over the full observation window and define its positive activity weight as $w_{j}=1+ln(1+a_{j})$. The base selection probability is

(1) $$q_{ij}=\frac{1-\alpha }{i}+\alpha \frac{{\left(k_{j}+1\right)}^{\beta }{w_{j}}^{\theta }}{\sum_{l=0}^{i-1}{\left(k_{l}+1\right)}^{\beta }{w_{l}}^{\theta }}$$

In Equation (1), $q_{ij}$ is the base probability for candidate j, α is the mixture coefficient in [0, 1], β is a degree switch taking values 0 or 1, and θ is the nonnegative activity exponent. The summation index l ranges over the same i older candidates. The added unit in $k_{j}+1$ ensures positive weights for isolated nodes. Both terms are normalized over this candidate set, so their mixture sums to one. The denominator i is therefore consistent with zero-based indexing; a one-based convention would require i−1.

The arriving node selects $m_{i}$ distinct targets without replacement. Degrees and base probabilities are frozen for the whole arrival. For draw r, let $U_{r}$ be the set of previously selected targets, with $U_{1}$ empty, and let $J_{r}$ denote the next selected target. Selection from the remaining candidates follows

(2) $$Pr(J_{r}=j|U_{r})=\frac{q_{ij}}{\underset{l\notin Uᵣ}{\sum }q_{il}},\;\;\;\;\;j\notin U_{r},\;\;\;\;\;r=1,\ldots ,m_{i}$$

The sum in Equation (2) is restricted to older candidates not in $U_{r}$; selected candidates have conditional probability zero. This is sequential weighted sampling without replacement, implemented by NumPy 2.3.5 choice with replace = False. The base $q_{ij}$ is not the marginal inclusion probability when several targets are drawn. All selected edges are added together, after which the arriving node and its targets receive their degree increments. Degrees are not updated between draws. If $m_{i}=0$, no target is drawn; if $m_{i}=i$, every older node is selected. Appendix A gives the complete algorithm.

Table A1 in Appendix A lists the five principal parameter combinations.

The five settings isolate uniform allocation, degree reinforcement, empirical activity preference, their combination, and a uniform-combined mixture. We replace the ambiguous label targeted growth with combined attachment to distinguish a growth mechanism from a node-removal attack. The hybrid value 0.55 has no privileged empirical status; interpretation rests on the broader parameter analysis rather than on this single setting.

### 2.3. Node-Removal Protocols

Random failure uses a uniformly shuffled permutation of all nodes. Static degree attack orders nodes by descending intact-network degree. Static betweenness attack orders them by exact intact-network shortest-path betweenness, defined as the sum over unordered endpoint pairs of the fraction of shortest paths passing through a node, excluding the endpoints [15,16]. Unreachable pairs contribute zero. Ranking does not depend on the common normalization factor. Static rankings are fixed before the first deletion and are not recomputed.

Adaptive degree attack recalculates the degree of every surviving node after each deletion and removes the current highest-degree node. For all centrality-based attacks, a seeded random permutation supplies a fixed secondary priority to break equal scores; the same priorities are used across attacks on a given graph. This prevents node identifier or arrival order from systematically resolving ties. Counterfactual confidence intervals include one independently seeded priority per realization, but observed targeted trajectories are conditional on their reported fixed priority and have no empirical-population confidence interval.

We additionally test batch-adaptive exact betweenness on the two observed graphs. At each successive removal-grid boundary, betweenness is recomputed on the surviving graph, and the next batch is removed in descending order. The batch size is the difference between consecutive rounded grid counts, which is approximately 2.5% of the original nodes. This diagnostic is not a one-node-at-a-time betweenness attack and is not extended to the full counterfactual ensemble. Network dismantling, feature-dependent percolation, and modular cascades motivate examining more than one attack protocol, but they are not simulated here [17,18,19].

Connectivity is evaluated after each deletion internally. Reported curves are sampled at nominal fractions p=0,0.025,...,0.95, using the nearest integer to pN (ties to even). Thus, the realized fraction differs from the nominal grid by at most half a node divided by N. No rewiring, edge repair, dynamical failure cascade, or node recovery is allowed.

### 2.4. Structural Outcomes and Uncertainty

For a removed fraction p, let $C_{1}(p)$ be the largest surviving connected component and let $C_{l}(p)$, for l=2,...,L(p), denote the remaining components; L(p) is their total count. If the largest components tie, exactly one is excluded from the finite-component sums, which leaves the sums unchanged. The largest-component ratio is

(3) $$S(p)=\frac{\left|C_{1}(p)\right|}{N}$$

Equation (3) uses the original node count rather than the number of survivors. Therefore, S(p) measures the retained connected fraction of the original system and cannot exceed the surviving-node fraction. Finite-cluster susceptibility is

(4) $$\chi (p)=\frac{\sum_{l=2}^{L(p)}{\left|C_{l}(p)\right|}^{2}}{\sum_{l=2}^{L(p)}\left|C_{l}(p)\right|}$$

In Equation (4), vertical bars denote component size. When no node remains outside the selected largest component, the denominator is zero and χ(p) is defined as zero. Let P denote the stated removal grid. We retain a fragmentation-peak location only when the positive global susceptibility maximum is unique and strictly interior to P:

(5) $$p_{c}=\underset{p\in P}{argmax}\chi (p)$$

Equation (5) is subject to this uniqueness and interior-maximum condition. Boundary maxima and plateaus are recorded separately and yield n.d. (not determined), not a substituted endpoint. Numerically equal maxima are identified with relative tolerance 10^−10^ and absolute tolerance 10^−12^. For random failure, the peak is found on the mean susceptibility curve across 50 deletion orders for that graph; it is not the mean of individual-sequence peak locations. The same peak-selection rule applies to all attacks. We do not exclude random-failure peaks by construction.

The curve-integrated robustness index is

(6) $$RI=\frac{1}{p_{max}}\int_{0}^{pₘₐₓ}S(p)dp,\;\;\;\;\;p_{max}=0.95$$

Equation (6) is evaluated by the trapezoidal rule on the nominal grid, where $p_{max}$ is the largest evaluated fraction. The division by $p_{max}$ averages the retained connected fraction over the removal interval; it does not force the best possible curve to have value one. The point summary S(0.20) is also retained in the data archive. Unlike a single peak location, RI uses the entire observed deletion response.

Let $P_{k}$ be the fraction of intact-network nodes with degree k and K be the number of occupied degree classes, including degree zero when present. The normalized degree-class entropy is

(7) $$H=-\frac{\underset{k:Pₖ>0}{\sum }P_{k}lnP_{k}}{lnK}$$

Equation (7) is set to zero when K=1. Because K can vary between graphs, both the class probabilities and the normalization can affect H. The maximum degree is the largest node degree; the degree Gini coefficient measures inequality across individual nodes. We also report the mean local clustering, assigning zero to nodes with degree less than two. These descriptors are evaluated on intact graphs only.

Intact-network global efficiency follows the average inverse shortest-path definition [20]:

(8) $$E_{0}=\frac{1}{N(N-1)}\underset{u\neq v}{\sum }\frac{1}{d_{uv}}$$

In Equation (8), u and v are distinct nodes, and $d_{uv}$ is their unweighted undirected shortest-path distance; disconnected pairs contribute zero. The sum is over ordered pairs. We compute $E_{0}$ exactly from all source nodes, not from sampled sources. Efficiency describes baseline accessibility and is not used as an outcome for attacked networks.

All counterfactual settings use n=30 independent graph realizations. For random failure, 50 independent deletion permutations are averaged within each graph before computing the between-graph uncertainty. For any realization-level metric x, the reported mean and two-sided 95% confidence interval are

(9) $$\bar{x}\pm t_{0.975,n-1}\frac{s_{x}}{\sqrt{n}}$$

In Equation (9), n is the number of independent observations entering a particular summary, the overbar denotes their arithmetic mean, and $s_{x}$ is their sample standard deviation, calculated with divisor n−1. The standard error of the mean is $\frac{s_{x}}{\sqrt{n}}$; it is distinct from the sample standard deviation. The multiplier $t_{0.975,n-1}$ is the 97.5th percentile of a Student t distribution with n−1 degrees of freedom. A two-sided 95% interval uses this percentile without doubling the observation count.

For counterfactual summaries, n=30 graph realizations are the independent observations. The 50 random deletion orders are averaged within each graph before estimating the between-graph standard error; they do not constitute 1500 independent graph observations. For random failure on an observed network, n=50 deletion orders quantify uncertainty conditional on that one topology. Paired attack comparisons use 30 graph-level differences. Peak intervals use the number of valid peaks, which can be smaller than 30; no interval is assigned when fewer than two valid observations are available. Curve bands apply Equation (9) pointwise and are not simultaneous bands. These Monte Carlo intervals do not quantify uncertainty about a population of empirical networks. Supplementary Section S3 gives the corresponding uncertainty conventions.

For peak locations, summaries condition on realizations with a valid peak and always report the valid fraction. A single valid peak has no estimated interval; no valid peak is reported as n.d. These intervals describe Monte Carlo variation under fixed empirical constraints, not uncertainty about the broader population of communication networks. Static-to-adaptive differences are paired within the same graph. Cross-kernel comparisons are not treated as paired because their random streams differ.

### 2.5. Sensitivity and Numerical Checks

With degree and activity both enabled, the mixing coefficient α is evaluated at 0, 0.1, ..., 1, supplemented by the main hybrid setting 0.55. The activity exponent θ is evaluated at 0, 0.5, 1, 1.5, and 2 for α=0.55 and 1, with β=1. Each point uses 30 realizations and static degree attack; these sweeps are not an exhaustive attack-by-parameter factorial experiment. Reused settings are cached rather than counted as new realizations. The complete analysis contains 1320 counterfactual graph instances and two observed graphs; 300 counterfactuals receive the full main attack suite.

The main betweenness attacks now use exact scores. To quantify the previous approximation, we compare 64, 128, and 256 sampled Brandes sources with exact scores on each observed graph and the first realization of each of the five principal kernels. Ten nested source samples are used at each size. Spearman score correlation, overlap of the highest-ranked 10% of nodes, and the absolute resulting RI error assess different consequences of approximation. This 12-graph diagnostic is not an ensemble-wide guarantee.

For all the main graphs, static-degree outcomes are reevaluated on a finer grid with a step of 0.005 without changing the underlying deletion order. Prefix comparisons use 10, 20, and 30 graph realizations and 20 versus 50 random orders. Numerical implementations of betweenness, efficiency, connected components, and susceptibility are checked against NetworkX 3.5 on three independent small test graphs. Supplementary Section S1–S7 report the design, provenance, dispersion, approximation, grid, convergence, and software details, respectively. These checks assess computational stability rather than external validity. The analyses used Python 3.13.9 with NumPy 2.3.5, pandas 2.3.3, SciPy 1.16.3, NetworkX 3.5, numba 0.62.1, and Matplotlib 3.10.6.

## 3. Results

### 3.1. Degree and Activity Have Distinct Structural Effects

**Figure 2:**
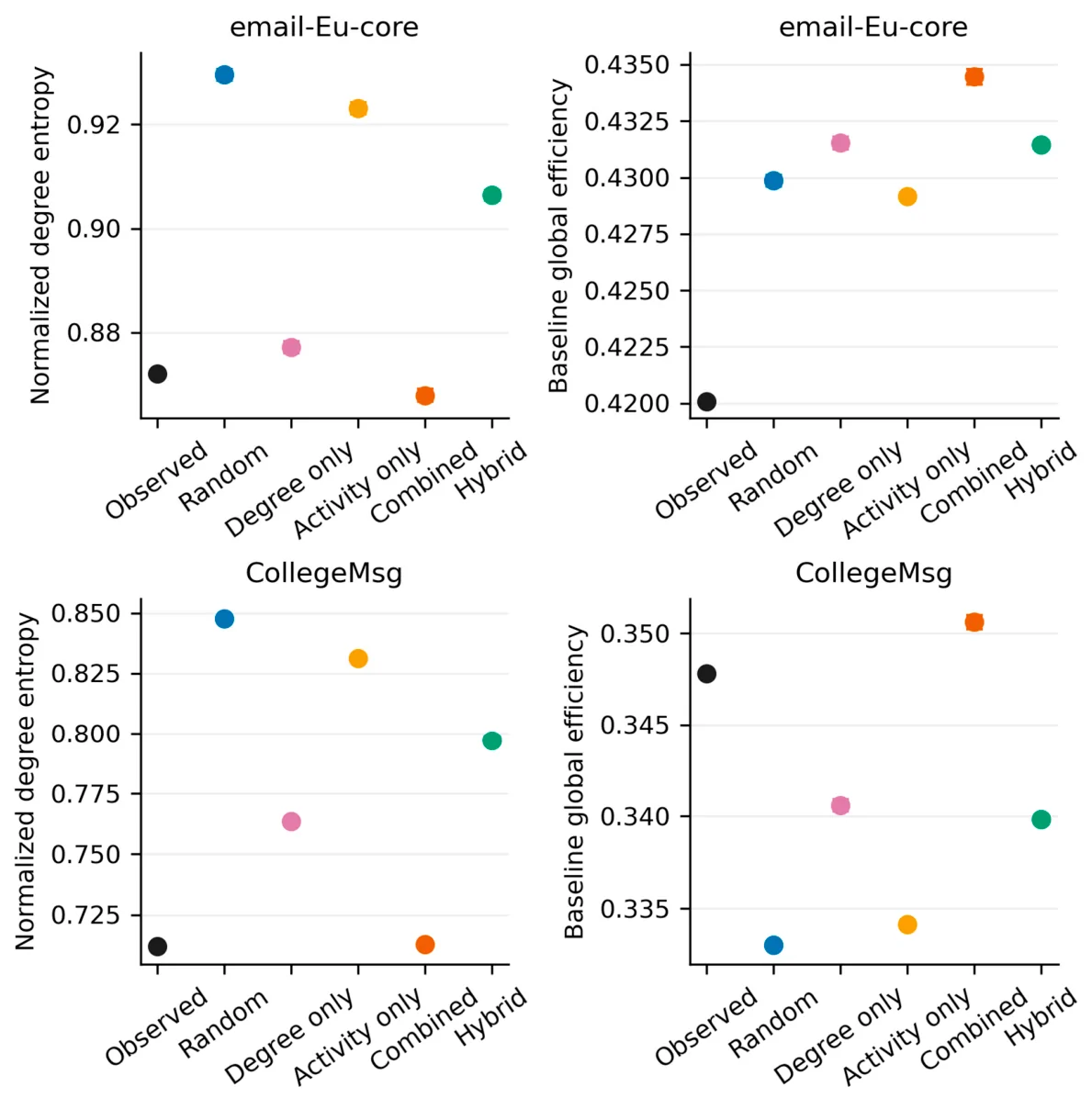
Intact-network degree-class entropy and exact global efficiency. Points and error bars show means and 95% confidence intervals across 30 graph realizations. Observed points describe a single graph and have no graph-ensemble error bar.

The counterfactual kernels produce different degree organizations despite identical arrival budgets. Figure 2 and Table 2 show that the combined kernel has lower degree-class entropy, higher degree inequality, and higher baseline efficiency than uniform attachment in both data sets. For email-Eu-core, mean degree Gini increases from 0.429 to 0.561; for CollegeMsg, it increases from 0.440 to 0.655. These node-level inequality changes support the concentration interpretation, which would not follow from entropy alone.

The activity-only model is not equivalent to preferential degree attachment. Its mean entropy remains 0.923 for email-Eu-core and 0.831 for CollegeMsg, compared with 0.877 and 0.764 for the degree-only model. Combining both factors raises the mean maximum degree beyond either single-factor kernel: to 260.3 for email-Eu-core and 248.1 for CollegeMsg. However, the observed graphs retain features outside this mechanism; for example, the observed email-Eu-core has a maximum degree of 345 and lower baseline efficiency than every counterfactual mean.

The efficiency gain from combined over uniform attachment is modest in email-Eu-core, from 0.430 to 0.434, and larger in CollegeMsg, from 0.333 to 0.351. Activity-only attachment does not universally improve efficiency over uniform attachment. The evidence therefore supports a specific property of the combined kernel in these data, not a general law that any form of heterogeneous preference improves accessibility.

### 3.2. Static Attacks and Mechanism Ablation

**Figure 3:**
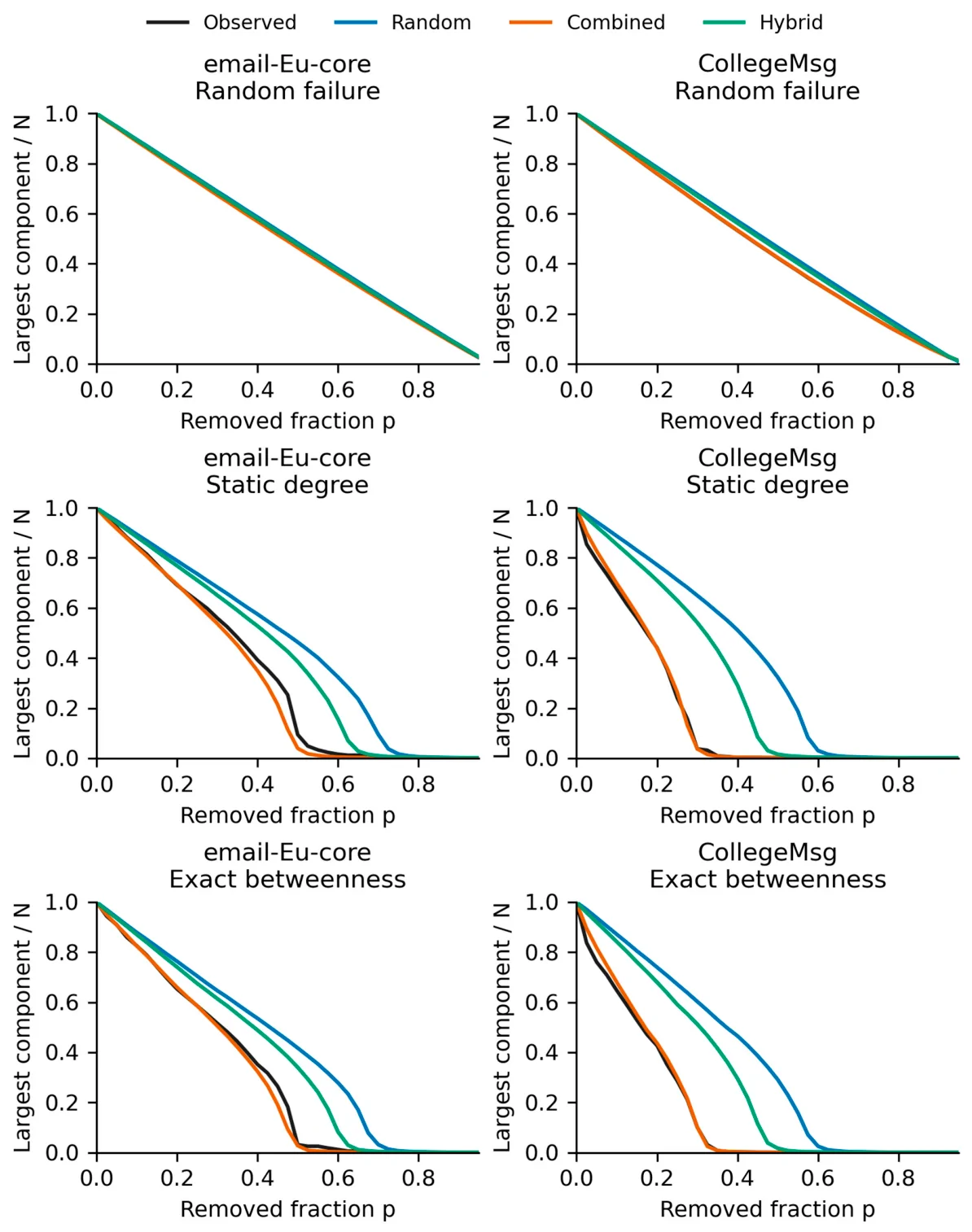
Largest-component curves for the observed network and three principal kernels. Counterfactual lines and bands are realization means and pointwise 95% confidence intervals, *n* = 30. Random-failure curves first average 50 deletion orders per graph; the observed random-failure band summarizes its 50 orders. Observed targeted curves are single fixed-priority trajectories. Static betweenness is exact.

Figure 3 compares random failure, static degree removal, and exact static betweenness removal. Table 3 adds all five kernels and adaptive degree outcomes. Under static degree attack, mean RI decreases from 0.456 for uniform attachment to 0.313 for the combined kernel in email-Eu-core, a relative reduction of 31.3%. The corresponding CollegeMsg values are 0.388 and 0.176, a reduction of 54.5%. The combined kernel is also less robust than either single-factor kernel.

Single-factor ablations separate the contributions that were confounded in the combined kernel. Static-degree RI for degree-only versus activity-only attachment is 0.338 versus 0.423 for email-Eu-core and 0.236 versus 0.330 for CollegeMsg. Thus, degree reinforcement produces a larger loss relative to uniform attachment than activity weighting alone under this parameterization. Multiplying the two factors reduces the robustness further, but this descriptive comparison is not a formal causal decomposition or a fitted interaction model.

Random failure is less destructive than intentional removal, but it does not reverse the uniform-versus-combined ranking. The mean random-failure RI is 0.510 versus 0.497 for email-Eu-core and 0.495 versus 0.467 for CollegeMsg. The differences between some intermediate rules are small, especially for email-Eu-core. Therefore, the results do not support a universal random error advantage for the most concentrated counterfactual. The attack-dependent change concerns the magnitude of vulnerability, not an obligatory reversal of rank.

### 3.3. Adaptivity Changes Magnitudes but Not the Main Kernel Ordering

**Figure 4:**
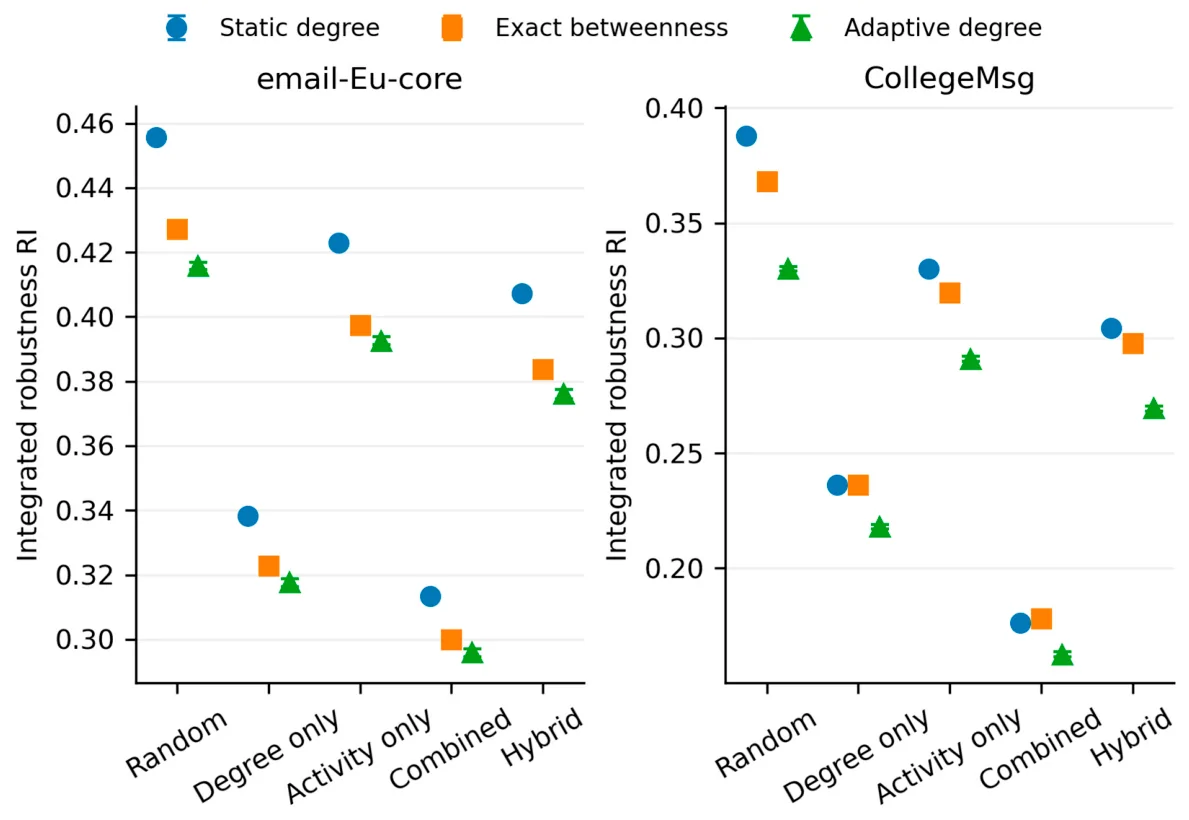
Integrated robustness under static degree, exact static betweenness, and one-node-adaptive degree attacks. Error bars are realization-level 95% confidence intervals, *n* = 30, with common graph instances across attack protocols.

Under static degree, exact static betweenness, and adaptive degree attacks, the mean counterfactual ordering in both data sets is random, activity only, hybrid, degree only, and then combined, from highest to lowest robustness. Figure 4 shows that recomputing degree after every deletion lowers RI for every kernel. This is stronger evidence than merely comparing two static centrality rankings because it allows the attacker to respond to the surviving topology.

The paired adaptive-minus-static degree difference for uniform attachment is −0.0400 [95% CI −0.0411, −0.0388] for email-Eu-core and −0.0578 [−0.0586, −0.0569] for CollegeMsg. For the combined kernel, it is −0.0175 [−0.0183, −0.0167] and −0.0138 [−0.0145, −0.0132], respectively. Adaptivity therefore changes the size of the gap between kernels even when their order remains unchanged. Supplementary Table S3 reports the paired differences for all kernels.

On the observed graphs, batch-adaptive exact betweenness reduces RI from the static value 0.310 to 0.288 for email-Eu-core and from 0.171 to 0.156 for CollegeMsg. These two diagnostics show sensitivity to betweenness updates but do not establish counterfactual ranking invariance under fully adaptive betweenness. The broader conclusion is restricted to the attack protocols actually evaluated.

### 3.4. Mixing and Activity Sensitivity

**Figure 5:**
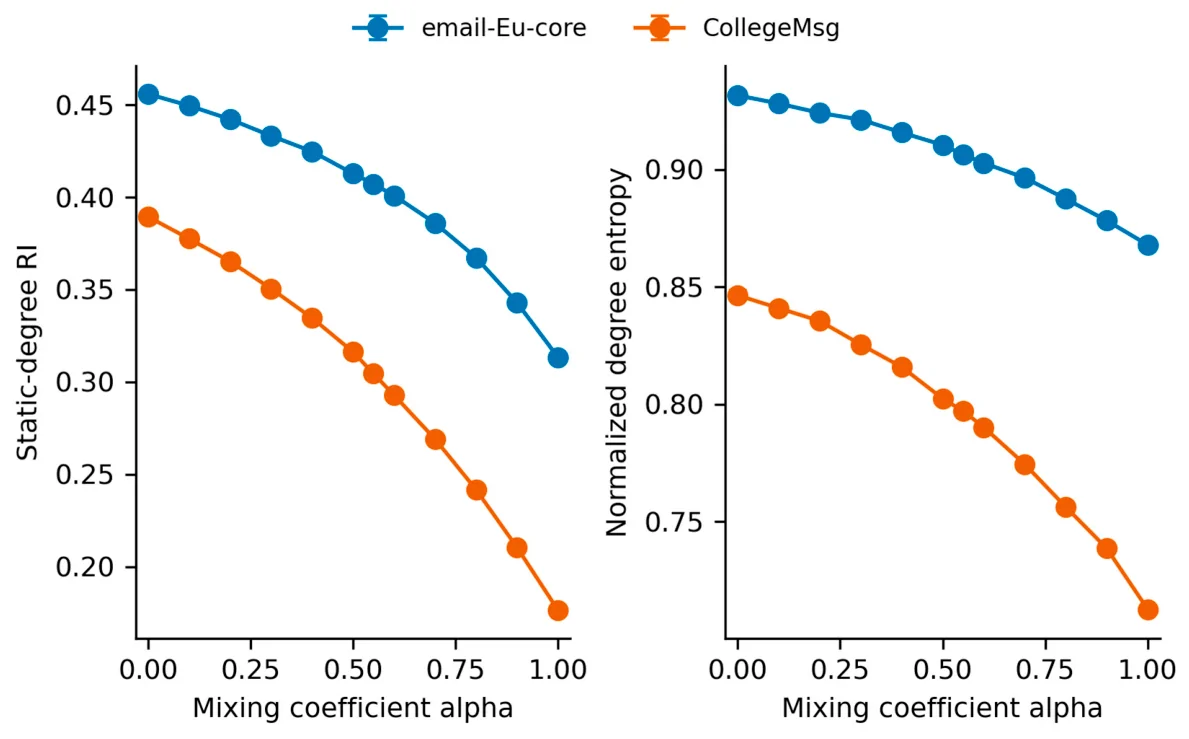
Mixing-coefficient sensitivity with degree and activity enabled. Each point uses 30 realizations; bars are 95% confidence intervals. The 0.55 point is included alongside the 0.1-spaced grid. Lines connect evaluated settings and do not imply a fitted continuous law.

Figure 5 replaces a three-point comparison with an explicit sweep of the mixture coefficient. Increasing the combined-preference share lowers the mean static-degree robustness across the evaluated coefficient grid in both data sets. Entropy changes in the same broad direction, but it is a correlated descriptor rather than a demonstrated causal mediator. The hybrid point 0.55 belongs to this continuum and was not selected by optimizing robustness or efficiency.

**Figure 6:**
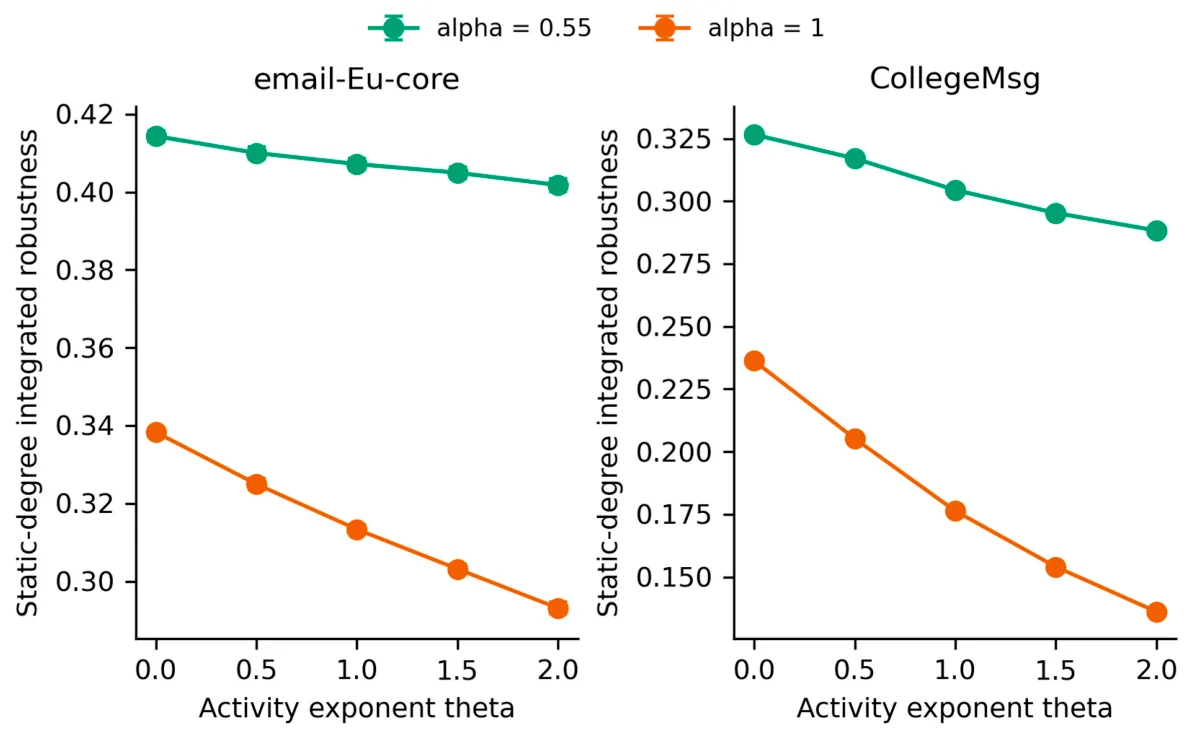
Activity exponent sensitivity for the combined and hybrid mixture coefficients. The degree switch is one; each setting uses 30 realizations. Error bars are 95% confidence intervals. The exponent-zero endpoint removes the activity factor without removing degree preference.

Figure 6 varies the activity exponent while keeping the degree factor enabled. The exponent-zero endpoint removes activity dependence. At full preference, this endpoint is precisely the degree-only model. Increasing the activity exponent from zero to two lowers the mean static-degree RI for both mixture settings in the two data sets. The direction of the main comparison is therefore not confined to the original activity exponent of one. This remains a finite parameter range result, not a claim about every possible activity transformation.

These sweeps clarify why the activity exponent is retained in the revised model: it now controls an evaluated source of variation. The degree-only and activity-only models provide the mechanism comparisons, while the exponent sweep tests the strength of one factor within the combined family. Neither analysis estimates actual decision rules used by individual participants.

### 3.5. Exact Centrality and Approximation Error

**Figure 7:**
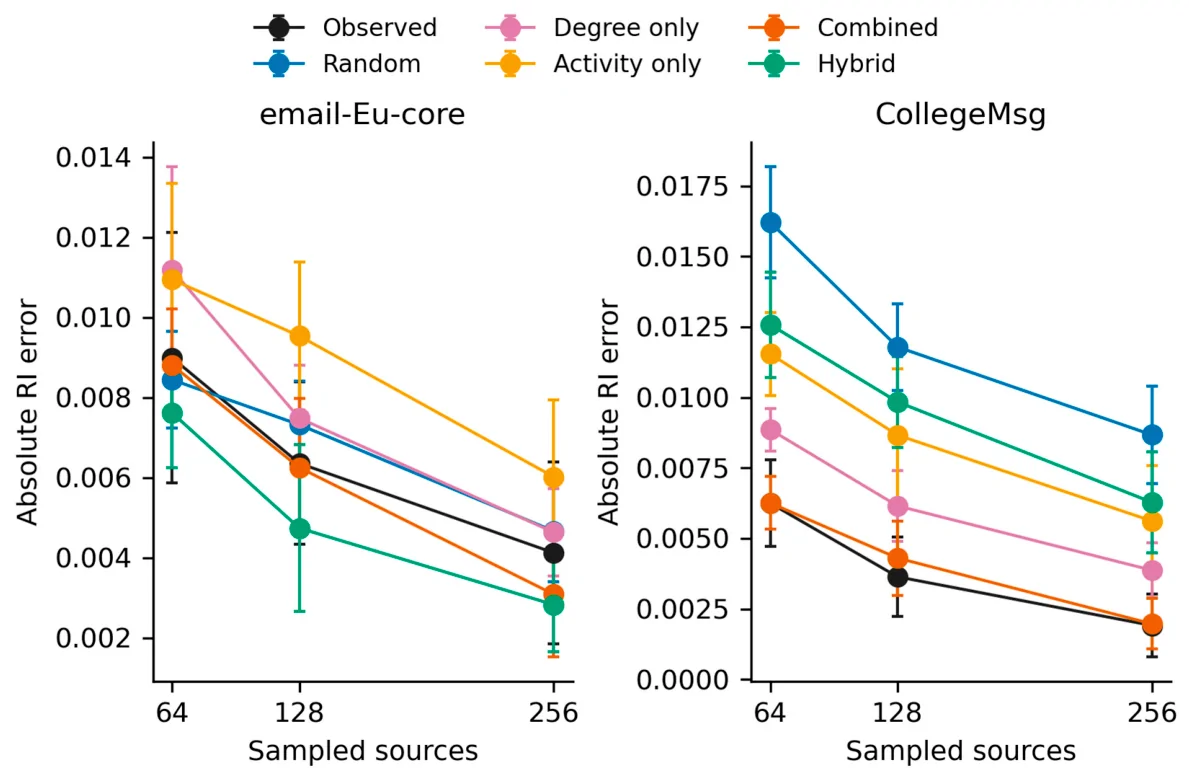
Absolute robustness error from source-sampled versus exact static betweenness. For each data set, one observed graph and the first realization of each kernel are tested. Points and bars show means and sample standard deviations across ten source samples for that fixed graph, not graph-ensemble confidence intervals. Source sets are nested across counts within each trial.

Figure 7 evaluates the effect of sampling Brandes sources. Averaged descriptively over the six diagnostic graphs and ten samples per graph, 64-source betweenness produces absolute RI errors of 0.0093 for email-Eu-core and 0.0103 for CollegeMsg. With 256 sources, these fall to 0.0042 and 0.0047. The largest 64-source error is 0.0190. These errors are not negligible compared with the differences between some intermediate kernels.

High score correlation does not imply an identical attack trajectory. At 64 sources, the mean Spearman correlations are approximately 0.955 and 0.961, whereas the overlap of the top-ranked 10% is only 0.719 and 0.733. Nodes with similar scores can exchange positions and alter the subsequent fragmentation curve. All main betweenness results therefore use exact scores; the sampled calculations are retained only as an approximation diagnostic.

### 3.6. Fragmentation Peaks and Grid Dependence

**Figure 8:**
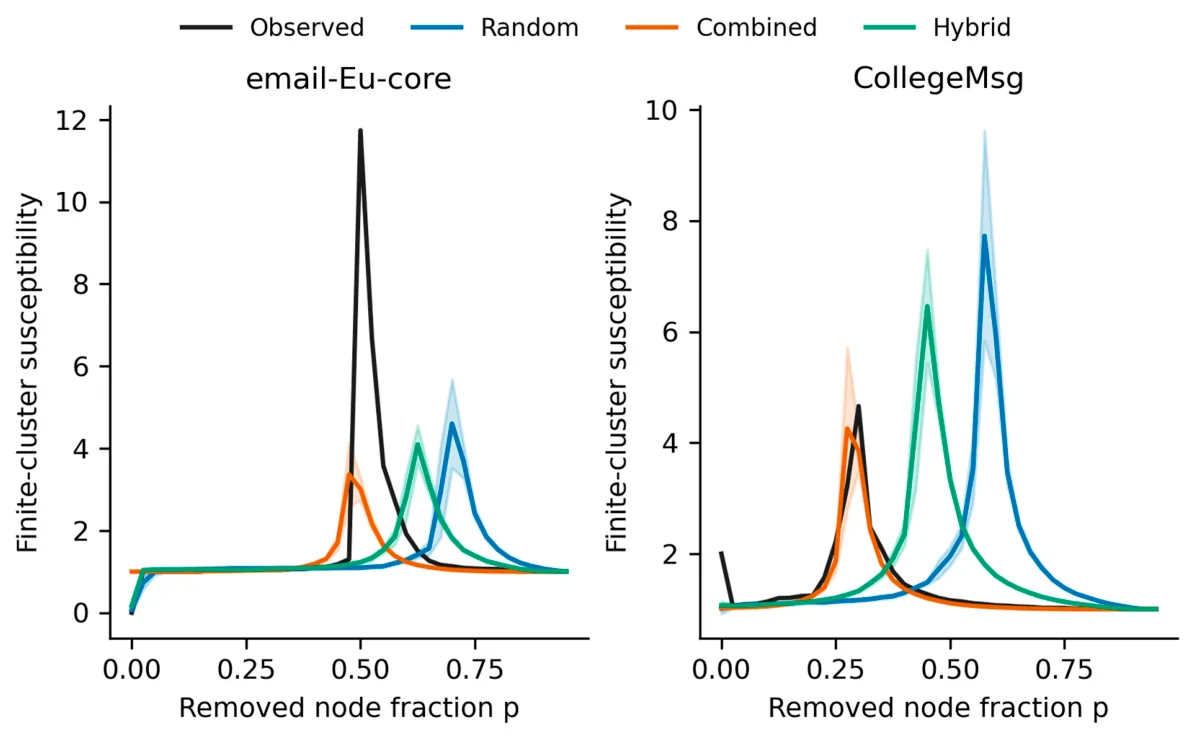
Finite-cluster susceptibility under static degree removal. Counterfactual lines and bands show means and pointwise 95% confidence intervals across 30 realizations. Observed curves are single trajectories. Peak summaries in Table 4 are calculated separately per realization, not from the plotted ensemble mean.

Figure 8 and Table 4 summarize susceptibility under static degree deletion. For the combined versus uniform kernels, the conditional mean peak fractions are 0.489 versus 0.708 for email-Eu-core and 0.292 versus 0.583 for CollegeMsg. Combined preference thus brings the finite-network fragmentation peak to a smaller removed fraction under this operational definition. A peak location should nevertheless be read alongside the full curve and integrated robustness.

The same peak rule occasionally detects a random-failure peak at 0.925: in 7 of 30 uniform, 1 of 30 activity-only, 2 of 30 combined, and 3 of 30 hybrid CollegeMsg graphs, and in 1 of 30 hybrid email-Eu-core graphs. Such sparse, near-boundary detections are insufficient to support a stable random-failure threshold. Most other random-failure curves have a boundary maximum. Missing peak entries describe this operational criterion, not missing simulations.

Refining the static-degree grid from 0.025 to 0.005 changes RI by at most 0.00144 across the 302 main graphs. By contrast, paired valid peak locations can move by as much as 0.070, and the one email-Eu-core hybrid plateau on the coarse grid becomes a valid peak on the finer grid. A narrow confidence interval across realizations cannot remove this discretization sensitivity. We therefore refer to finite-network fragmentation peaks and do not infer critical exponents, discontinuity, or a thermodynamic phase-transition point.

## 4. Discussion

The main empirical constraint is the arrival-specific link budget. Within that constraint, degree preference and activity preference produce distinguishable structural changes, and their combination yields the lowest intentional-attack robustness among the evaluated kernels. This supports an attribution to the specified degree-activity mechanism, not to preferential attachment in general. The single-factor comparisons also show that activity weighting is not merely a relabeling of degree reinforcement.

Baseline accessibility and deletion robustness answer different questions. A compact intact graph can lose connectivity quickly if its shortest paths and links depend on a small central set. In these data, the combined kernel improves exact baseline efficiency relative to uniform attachment but performs worse under both random and intentional deletion. The random-failure penalty is smaller; its existence rules out presenting random and targeted attack rankings as necessarily opposite.

Adaptivity makes the threat model explicit. A fixed centrality ordering represents an attacker acting on an initial map; adaptive degree removal represents repeated observation of the surviving graph. Both are legitimate scenarios, but they are not interchangeable. The stable kernel ordering under the latter strengthens the principal comparison, while the larger loss for some less concentrated graphs shows that absolute robustness values depend on the attack policy. The observed-only batch-betweenness check is suggestive rather than exhaustive.

Entropy is informative when accompanied by the distribution it summarizes. Here, lower degree-class entropy co-occurs with higher node-degree Gini and lower intentional-attack robustness along the combined-kernel sweep. These associations do not establish entropy as an independent design objective or a universal early-warning statistic. They identify a pattern within two constrained ensembles, whose mechanisms can be tested more directly by the ablation experiments.

The observed networks are not expected to be reproduced by any one kernel. Community membership, reciprocity, aging, and event timing are omitted. Similar scalar metrics can conceal very different connection patterns, as illustrated by CollegeMsg, whose observed entropy and Gini resemble the combined means. Appendix B explains why this resemblance does not validate the kernel as a behavioral model. Practical design implications should therefore be stated conditionally: distributing attachment opportunities can reduce the tested centrality-attack vulnerability, but operational cost and temporal reachability require separate analysis.

## 5. Limitations

The two data sets are communication networks, not independent samples of all growing networked systems. Their observation windows, incomplete visibility, and undirected aggregation constrain interpretation. Full-window activity and older-neighbor budgets incorporate future information relative to an online arrival process. The analysis is therefore unsuitable for causal claims about actual formation decisions or prospective robustness forecasts without a new time-respecting design.

The confidence intervals quantify Monte Carlo error conditional on each data set and model. They do not include uncertainty from data collection, alternative activity transformations, or unmodeled mechanisms. The parameter sweeps use static degree attack only; fully adaptive exact betweenness is not evaluated across the ensembles. Observed targeted curves are conditional on one tie-breaking priority. A separate tie-priority ensemble for each observed graph would be needed to quantify that source of uncertainty.

Finally, finite susceptibility peaks can be broad, tied, or grid-dependent. No finite-size scaling across independent network sizes is performed, so the work cannot establish a thermodynamic phase transition. Structural connectivity is not a measure of dynamic recovery, functional service quality, or system-wide adaptive resilience. The conclusions remain limited to undirected node-removal robustness under the stated retrospective constraints.

## 6. Conclusions

Holding empirical node order and arrival-specific link budgets fixed reveals a reproducible ordering within the evaluated counterfactual ensembles: combined degree-activity preference produces lower intentional-attack robustness than either single-factor preference, and uniform attachment gives the highest mean robustness under the three tested centrality-based protocols. Recalculating the degree after every deletion lowers robustness without changing that ordering. The combined kernel improves baseline efficiency relative to uniform attachment but does not gain a random-failure advantage in these two data sets. Activity and mixing sweeps support the direction of the principal comparison, whereas the sensitivity of susceptibility peaks to grid refinement limits claims about criticality. The evidence concerns structural robustness of finite, retrospectively constrained networks, not dynamical recovery or a universal phase-transition law.

## Figures and Tables

**Table 1:** Empirical temporal data after self-loop removal.

Data Set	Nodes N	Edges M	Events	Duration (Days)
email-Eu-core	986	16,064	332,334	803.93
CollegeMsg	1899	13,838	59,835	193.71

Edges are unique undirected dyads; events retain repeated interactions. Duration is the difference between the first and last retained timestamps.

**Table 2:** Topology under the five attachment kernels.

Data Set	Rule	Degree Entropy H	Degree Gini	Baseline $E_{0}$
email-Eu-core	Observed	0.872	0.544	0.4201
	Random	0.930 [0.928, 0.931]	0.429 [0.428, 0.429]	0.4299 [0.4296, 0.4301]
	Degree only	0.877 [0.876, 0.878]	0.543 [0.543, 0.544]	0.4315 [0.4313, 0.4318]
	Activity only	0.923 [0.922, 0.924]	0.453 [0.452, 0.453]	0.4292 [0.4290, 0.4293]
	Combined	0.868 [0.867, 0.869]	0.561 [0.560, 0.562]	0.4345 [0.4341, 0.4348]
	Hybrid	0.906 [0.905, 0.907]	0.485 [0.484, 0.486]	0.4315 [0.4313, 0.4316]
CollegeMsg	Observed	0.712	0.653	0.3478
	Random	0.848 [0.846, 0.849]	0.440 [0.439, 0.441]	0.3330 [0.3329, 0.3331]
	Degree only	0.764 [0.762, 0.765]	0.596 [0.595, 0.597]	0.3406 [0.3403, 0.3409]
	Activity only	0.831 [0.830, 0.833]	0.501 [0.500, 0.501]	0.3341 [0.3339, 0.3344]
	Combined	0.713 [0.711, 0.714]	0.655 [0.654, 0.656]	0.3506 [0.3502, 0.3510]
	Hybrid	0.797 [0.795, 0.799]	0.533 [0.533, 0.534]	0.3398 [0.3396, 0.3401]

Values are means [95% confidence intervals], *n* = 30, except for single observed graphs. H is normalized degree-class entropy; Gini is node-degree inequality; $E_{0}$ is exact intact-network global efficiency. All three metrics are dimensionless.

**Table 3:** Integrated structural robustness by attack protocol.

Data Set	Rule	Random Failure	Static Degree	Static Betweenness	Adaptive Degree
email-Eu-core	Observed	0.4998 [0.4988, 0.5008]	0.3316	0.3098	0.3074
	Random	0.5098 [0.5096, 0.5100]	0.4558 [0.4544, 0.4571]	0.4273 [0.4257, 0.4288]	0.4158 [0.4147, 0.4169]
	Degree only	0.4984 [0.4981, 0.4986]	0.3383 [0.3370, 0.3395]	0.3227 [0.3214, 0.3240]	0.3176 [0.3164, 0.3188]
	Activity only	0.5050 [0.5048, 0.5052]	0.4229 [0.4216, 0.4241]	0.3974 [0.3959, 0.3988]	0.3926 [0.3914, 0.3937]
	Combined	0.4972 [0.4969, 0.4974]	0.3133 [0.3121, 0.3146]	0.3001 [0.2988, 0.3013]	0.2958 [0.2945, 0.2972]
	Hybrid	0.5055 [0.5053, 0.5056]	0.4072 [0.4057, 0.4086]	0.3838 [0.3821, 0.3854]	0.3761 [0.3746, 0.3775]
CollegeMsg	Observed	0.4672 [0.4652, 0.4692]	0.1723	0.1707	0.1533
	Random	0.4951 [0.4948, 0.4953]	0.3879 [0.3865, 0.3892]	0.3680 [0.3667, 0.3694]	0.3301 [0.3292, 0.3310]
	Degree only	0.4758 [0.4754, 0.4761]	0.2363 [0.2351, 0.2375]	0.2361 [0.2350, 0.2372]	0.2180 [0.2171, 0.2189]
	Activity only	0.4885 [0.4882, 0.4887]	0.3300 [0.3285, 0.3314]	0.3196 [0.3183, 0.3209]	0.2910 [0.2900, 0.2920]
	Combined	0.4675 [0.4672, 0.4678]	0.1764 [0.1753, 0.1774]	0.1783 [0.1772, 0.1795]	0.1626 [0.1615, 0.1636]
	Hybrid	0.4876 [0.4874, 0.4878]	0.3044 [0.3031, 0.3058]	0.2978 [0.2965, 0.2991]	0.2693 [0.2684, 0.2703]

Entries are dimensionless RI means [95% confidence intervals]. Counterfactual intervals use 30 graph means; observed random-failure intervals use 50 orders. Observed intentional attacks give one value conditional on a fixed tie priority. Betweenness uses exact scores; adaptive degree updates after every deletion.

**Table 4:** Operational fragmentation peaks on the 0.025 removal grid.

Data Set	Rule	Static-Degree $p_{c}$	Valid/Total	Random-Failure $p_{c}$	Valid/Total
email-Eu-core	Observed	0.500	1/1	n.d.	0/1
	Random	0.708 [0.702, 0.715]	30/30	n.d.	0/30
	Degree only	0.527 [0.520, 0.533]	30/30	n.d.	0/30
	Activity only	0.646 [0.641, 0.651]	30/30	n.d.	0/30
	Combined	0.489 [0.483, 0.496]	30/30	n.d.	0/30
	Hybrid	0.626 [0.619, 0.633]	29/30	0.925	1/30
CollegeMsg	Observed	0.300	1/1	n.d.	0/1
	Random	0.583 [0.578, 0.589]	30/30	0.925 [0.925, 0.925]	7/30
	Degree only	0.362 [0.355, 0.368]	30/30	n.d.	0/30
	Activity only	0.488 [0.482, 0.494]	30/30	0.925	1/30
	Combined	0.292 [0.287, 0.297]	30/30	0.925 [0.925, 0.925]	2/30
	Hybrid	0.453 [0.447, 0.460]	30/30	0.925 [0.925, 0.925]	3/30

$p_{c}$ is a unique positive interior global susceptibility maximum. Values are conditional means [95% confidence intervals] for valid realizations. n.d. means no valid peak; a single valid realization has no confidence interval. Boundary and plateau counts are listed in Supplementary Table S2. Zero-width intervals can occur because the grid is discrete.

## Data Availability

The email-Eu-core temporal network and CollegeMsg data are publicly available from the Stanford Large Network Dataset Collection at https://snap.stanford.edu/data/email-Eu-core-temporal.html (accessed on 21 May 2026) and https://snap.stanford.edu/data/CollegeMsg.html (accessed on 21 May 2026). The input files analyzed in this study, their checksums, the numerical results, and the analysis and plotting code are included in the accompanying Data_and_Code.zip Supplementary archive.

## Supplementary Materials

The following supporting information can be downloaded at: https://www.mdpi.com/article/10.3390/e28101135/s1. The supplementary document contains S1 Analysis design and parameters; S2 Data provenance and integrity; S3 Dispersion, peak status, and paired attacks; S4 Betweenness approximation diagnostic; S5 Removal-grid diagnostic; S6 Monte Carlo prefix diagnostics; and S7 Computational implementation. Data_and_Code.zip contains the source data, graph-level and attack-level numerical results, analysis and plotting scripts, and the data dictionary.

## Abbreviations

The following abbreviations are used in this manuscript:

SNAP	Stanford Network Analysis Project
LCC	Largest connected component
RI	Integrated robustness index
CI	Confidence interval
SD	Sample standard deviation
n.d.	Not determined

## Appendix A

The attachment procedure used in Section 2.2 is stated below. Its inputs are the node order, the older-neighbor budgets, full-window activity weights, and the three kernel parameters. Table A1 gives the principal settings.

**Table A1:** Principal counterfactual kernels.

Rule	α	β	θ	Preferential Score
Random	0	0	0	Uniform selection
Degree only	1	1	0	$k_{j}+1$
Activity only	1	0	1	$w_{j}$
Combined	1	1	1	$(k_{j}+1)w_{j}$
Hybrid	0.55	1	1	$(k_{j}+1)w_{j}$

The hybrid coefficient 0.55 is an illustrative intermediate setting, not an estimated or optimized parameter. Parameter sweeps are described in Section 2.5.

Step 1. Initialize N isolated nodes, zero degrees, and an empty undirected edge set.
Step 2. For each arriving index i from 1 through N−1, read its fixed empirical budget $m_{i}$. If the budget is zero, advance to the next node.
Step 3. Freeze the current degrees of all older candidates and compute the normalized base probabilities in Equation (1). Initialize the selected target set as empty.
Step 4. Draw $m_{i}$ distinct targets using the conditional probabilities in Equation (2). Remove selected candidates from subsequent draws, but do not change the frozen degrees or base probabilities.
Step 5. Add all selected edges. Increase each selected target degree by one and the arriving node degree by $m_{i}$. Continue with the next arrival.
Step 6. Verify that each node has exactly its assigned number of older-endpoint edges and that the total edge count equals the empirical M.

This algorithm produces a simple undirected graph with neither self-loops nor parallel edges. The empty initial graph permits isolated early nodes; the positive degree offset makes them eligible targets. It does not require an arbitrary seed clique that would change the empirical link budget.

## Appendix B

The counterfactual estimand in Section 2.1 and Section 4 is the difference in structural outcomes induced by changing target selection while holding empirical node order, activity weights, and arrival budgets fixed. It does not estimate the causal effect of a real organizational intervention. Empirical activity is measured from the entire observation period and may itself be related to degree and community membership.

Accordingly, agreement between an observed scalar metric and a counterfactual mean is insufficient to identify a growth rule. Distinct models can share an entropy or efficiency value while differing in motifs, communities, directionality, and temporal reachability. Testing those properties would require additional observables and a separate validation design.

## References

[1] Newman M.E.J. (2003). The structure and function of complex networks. SIAM Rev..

[2] Albert R., Jeong H., Barabasi A.-L. (2000). Error and attack tolerance of complex networks. Nature.

[3] Cohen R., Erez K., ben-Avraham D., Havlin S. (2000). Resilience of the Internet to random breakdowns. Phys. Rev. Lett..

[4] Artime O., Grassia M., De Domenico M., Gleeson J.P., Makse H.A., Mangioni G., Perc M., Radicchi F. (2024). Robustness and resilience of complex networks. Nat. Rev. Phys..

[5] Liu X., Li D., Ma M., Szymanski B.K., Stanley H.E., Gao J. (2022). Network resilience. Phys. Rep..

[6] Li M., Liu R.-R., Lu L., Hu M.-B., Xu S., Zhang Y.-C. (2021). Percolation on complex networks: Theory and application. Phys. Rep..

[7] Pan X., Guo Z. (2025). Dynamic resilience in complex networks. Chaos Solitons Fractals.

[8] Proverbio D., Boccaletti S. (2026). Perspectives on robustness and resilience of complex networks. Entropy.

[9] Barabasi A.-L., Albert R. (1999). Emergence of scaling in random networks. Science.

[10] Bianconi G., Barabasi A.-L. (2001). Competition and multiscaling in evolving networks. Europhys. Lett..

[11] Paranjape A., Benson A.R., Leskovec J. Motifs in temporal networks. Proceedings of the Tenth ACM International Conference on Web Search and Data Mining.

[12] Panzarasa P., Opsahl T., Carley K.M. (2009). Patterns and dynamics of users’ behavior and interaction: Network analysis of an online community. J. Am. Soc. Inf. Sci. Technol..

[13] Wen X., Zhou J., Jiang S., Zhou Q., Lu J.-a. (2025). Dynamics resilience of complex networks under edge-additions. Chaos Solitons Fractals.

[14] Znaidi M.R., Sia J., Ronquist S., Rajapakse I., Jonckheere E., Bogdan P. (2023). A unified approach of detecting phase transition in time-varying complex networks. Sci. Rep..

[15] Freeman L.C. (1977). A set of measures of centrality based on betweenness. Sociometry.

[16] Brandes U. (2001). A faster algorithm for betweenness centrality. J. Math. Sociol..

[17] Grassia M., De Domenico M., Mangioni G. (2021). Machine learning dismantling and early-warning signals of disintegration in complex systems. Nat. Commun..

[18] Artime O., De Domenico M. (2021). Percolation on feature-enriched interconnected systems. Nat. Commun..

[19] Snyder J., Cai W., D’Souza R.M. (2022). Degree-targeted cascades in modular, degree-heterogeneous networks. Phys. Rev. Res..

[20] Latora V., Marchiori M. (2001). Efficient behavior of small-world networks. Phys. Rev. Lett..

